# Supplementary material for: Modelling and analysis of cAMP-induced mixed-mode oscillations in cortical neurons: Critical roles of HCN and M-type potassium channels
Source: PLoS Comput Biol. 2024 Mar 22;20(3):e1011559. doi: 10.1371/journal.pcbi.1011559 (PMC10990174; doi:10.1371/journal.pcbi.1011559)
Supplement: Attachment — Submitted filename: Modelling and analysis of cAMP - review LZ.pdf [file pcbi.1011559.s001.pdf]

Modelling and analysis of cAMP-induced mixed-mode oscillations in cortical neurons: Critical roles of HCN and M-type potassium channels

This paper proposes to investigate the effect of M-currents and hyperpolarization activated cyclic nucleotide-gated channels on various neuronal activation patterns and in particular on the emergence of mixed mode oscillations. The proposed model is a preexisting conductance-based model that has been adapted to include the specific currents the authors are interested in. The second part of the paper focuses on the mathematical analysis of the model, using the fact that it is a slow-fast dynamical system.

Altogether the paper is nice and well-written, the mathematical analysis of the model is quite extensive and allows to understand what are the various behaviors that can be reproduced by the model. The application part is interesting but it could benefit from a more precise quantification of the various behaviors the authors have reproduced to allow for a more direct comparison to the experimental results that they refer too. It would also be nice to show the plots of the various currents that are studied and discussed (not only the voltage) in order to have a better idea of what is going on (both in the model and also for the biological interpretation). More specifically:

Figures 2 to 5 and associated results sections:

- It would be interesting to add the quantifications of the various modified behaviors that you mention (augmentation of the hyperpolarization, change in firing rates, threshold values for apparition of MMOs etc.). This would allow for a more direct comparison to the experimental results that you mention all along. I noted that in the discussion you say that your model *qualitatively* reproduces the experimental results. Then, what I don't know is if it does or not reproduce them *quantitatively* too? Anyway, I think it's OK even if it does not match exactly the experimental results (then the possible reason.s why it doesn't should be quickly discussed) but it would be nice to have these quantifications at least to compare in between the model simulations..
- It would be very nice to also plot the various currents that you mention and that are modified for each scenario. This would give a better understanding of the possible biological mechanisms that are investigated here. Moreover this is what is announced first in the abstract and introduction and having a graphical illustration of these effects is much more intuitive than just having its written description in the results section.

Line 138: Can you quantify how much *less LAO and more SAO* is? You mention that this is because of the reduction from 5D to 3D (which makes sense) and it's always nice to have an idea of the effect of the approximations made.

Discussion line 298 you state that: *"We thus suggest that when neuronal and glial cells interact [4], both these two channels are involved to allow glial cells to finely tune the electrical behavior in individual neurons."* I think saying it just like that is a bit far-fetched.. It would be nice to add some context or explanation of the mechanisms you are thinking about to allow to better understand what you mean here (I think it's interesting but it feels like you skipped some reasoning steps here…).

Minor comments:

Line 55 : I would say *simulated* or *numerically* solved, rather than just solved.

Line 92: Could you add one sentence to state how the administration of Forksolin and Linopridine? Because you say one increases the gM while the other one reduces gM, so it's a bit unclear for me..

Figure 6: Could you add the legend in the graph too for the meaning of the various colors (I know it's in the caption but I think it would facilitate the understanding to have it graphically too).

Figure 8: The layout is a bit weird, having C above B makes it confusing.. Also can you explain the difference between left and right in panel B ? It's a bit unclear in the caption..
