# Supplementary material for: Modelling and analysis of cAMP-induced mixed-mode oscillations in cortical neurons: Critical roles of HCN and M-type potassium channels
Source: PLoS Comput Biol. 2024 Mar 22;20(3):e1011559. doi: 10.1371/journal.pcbi.1011559 (PMC10990174; doi:10.1371/journal.pcbi.1011559)
Supplement: Attachment — Submitted filename: PLoS_Answers_NeuronalModel.pdf [file pcbi.1011559.s002.pdf]

Point-to-pont responses

We wish to thank both reviewers for their excellent work and useful suggestions, which we believe helped us to improve the paper. We have now made all computer code (XPPAUT and MATLAB) available in the institutional repository of our university at `https://researchdata.cab.unipd.it/1180`.

In the following, we answer the points raised. All answers as well as changes to the manuscript are highlighted with the use of blue color.

Reviewer 1

This paper proposes to investigate the effect of M-currents and hyperpolarization activated cyclic nucleotide-gated channels on various neuronal activation patterns and in particular on the emergence of mixed mode oscillations. The proposed model is a preexisting conductance-based model that has been adapted to include the specific currents the authors are interested in. The second part of the paper focuses on the mathematical analysis of the model, using the fact that it is a slow-fast dynamical system.

Altogether the paper is nice and well-written, the mathematical analysis of the model is quite extensive and allows to understand what are the various behaviors that can be reproduced by the model. The application part is interesting but it could benefit from a more precise quantification of the various behaviors the authors have reproduced to allow for a more direct comparison to the experimental results that they refer too. It would also be nice to show the plots of the various currents that are studied and discussed (not only the voltage) in order to have a better idea of what is going on (both in the model and also for the biological interpretation). More specifically:

We appreciate the supporting comments a lot. We have addressed the issues raised in the point-to-point answers below.

1. It would be interesting to add the quantifications of the various modified behaviors that you mention (augmentation of the hyperpolarization, change in firing rates, thresholdvalues for apparition of MMOs etc.). This would allow for a more direct comparison to the experimental results that you mention all along. I noted that in the discussion you say that your model qualitatively reproduces the experimental results. Then, what I don't know is if it does or not reproduce them quantitatively too? Anyway, I think it's OK even if it does not match exactly the experimental results (then the possible reasons why it doesn't should be quickly discussed) but it would be nice to have these quantifications at least to compare in between the model simulations.

   As suggested, we have added the range of I_{app} values where MMOs are seen (lines 68, 91 and 108-110). Changes in firing frequency are provided in Fig. 6. We do

not mention “augmentation of hyperpolarization” anywhere in the manuscript, though the reviewer might refer to our description of the dynamics creating SAOs (line 69), which has now been made clearer by the introduction of the new Fig. 3 (as suggested by the reviewer in point 2 below). The main discrepancy between model results and the experimental findings regards the firing frequencies, which are too high in the model simulations. The same problem is evident in the simulations by Lezmy et al., but we decided to build upon their model as it was adapted to describe the pyramidal cells that they used for their experiments. In the Discussion, we now discuss this issue.

2. It would be very nice to also plot the various currents that you mention and that are modified for each scenario. This would give a better understanding of the possible biological mechanisms that are investigated here. Moreover this is what is announced first in the abstract and introduction and having a graphical illustration of these effects is much more intuitive than just having its written description in the results section.

   Thank you for this suggestion. We have now included a new Fig. 3 with the main currents (the ones with dynamic gating variables), and appropriate references to the figure in the main text (lines 70-77).

3. Line 138: Can you quantify how much less LAO and more SAO is? You mention that this is because of the reduction from 5D to 3D (which makes sense) and it’s always nice to have an idea of the effect of the approximations made.

   We now explicit state the number of SAOs and LAOs observed in the 3D and the 5D model simulations for $I_{App} = 250\ \mu\text{A/cm}^2$ (lines 165-167).

4. Discussion line 298 you state that: “We thus suggest that when neuronal and glial cells interact [4], both these two channels are involved to allow glial cells to finely tune the electrical behaviour in individual neurons.” I think saying it just like that is a bit far-fetched. It would be nice to add some context or explanation of the mechanisms you are thinking about to allow to better understand what you mean here (I think it’s interesting but it feels like you skipped some reasoning steps here...).

   We agree, the text was too concise. We now include (lines 356-365) a brief explanation of how glial cells activate A2aR by release of ATP, which in turn raises cAMP and activates HCN and M channel, and added a reference to Lezmy et al., who studied this pathwath in details. In addition, we provide examples of the physiological relevance of the control of neuronal firing frequency via MMOs by HCN and M channels.

5. Line 55 : I would say simulated or numerically solved, rather than just solved.

   We changed to "simulated" as suggested (line 62).

6. Line 92: Could you add one sentence to state how the administration of Forksolin and Linopridine? Because you say one increases the gM while the other one reduces gM, so it's a bit unclear for me..

   Indeed the text was not very clear. We have now rewritten the text (lines 118-121) and made a reference to Table 2 where the parameter values for the various scenarios are given explicitly. Hopefully it is now clear that Linopirdine reduces the M channel conductance in a FSK-stimulated cell (by 30%).

7. Figure 6: Could you add the legend in the graph too for the meaning of the various colors (I know it's in the caption but I think it would facilitate the understanding to have it graphically too).

   As suggested, we added the requested legend.

8. Figure 8: The layout is a bit weird, having C above B makes it confusing.. Also can you explain the difference between left and right in panel B ? It's a bit unclear in the caption..

   We changed the layout of the figure. Specifically, we merged the left and the right parts of the old panel B into the new panel A, which shows the bifurcation diagram under control conditions and I_{App} equal to 300 μA/cm^2. The two insets zoom on the areas near the PD$_1$ and the HB.

Reviewer 2

I was happy to see this paper appear as a submission to PLoS Computational Biology. I think it provides an excellent example of using ideas from the theory of multiple timescale dynamical systems to explain experimental observations in neural models that include the contributions of specific ion channels, and it is important to have this type of work disseminated to a broad audience such as that of PLoS CB. I do have a variety of comments aimed at improving how well the paper conveys its key points, at making it more suitable for the PLoS CB readership, and at making a few minor corrections. I will present these in order of appearance in the paper:

We appreciate these positive and supportive comments from reviewer a lot. We believe that the reviewer's thorough review and constructive suggestions and comments have helped us to improve the paper substantially.

1. Start of 1st section: Some clarification is needed: What types of neurons were used in the experiments in refs [4], [5]? What types of neurons is the model in this paper meant to describe? Do these match?

   We have now specified that the papers [4] and [5] used cortical pyramidal cells in their experiments in the second paragraph of the Introduction. The original model in [7] is, in contrast, a description of a motor nerve fibre. This model was adapted by Lezmy et al. to describe the data in [4], and we decided to use this modified model to agree with the choice made in [4], using the same set of adapted parameters, to reproduce the behaviour of a cortical neuron, see line 23 in the Introduction, line 50 in Results, and the beginning of the Materials and Methods.

2. Top of par 2 of 2nd section: Need some sort of reference ahead to the Methods so that readers know what is meant by "The system of ODEs". Also, please give a brief summary of the model components here, for readers who don't want to look ahead to Methods at this point.

   At the beginning of the Results section, we now briefly summarize the dynamical variables of the model. Moreover, we inserted a reference to the Materials and Methods section.

3. RMP is not a standard abbreviation for resting membrane potential. Better to use V_m.

   We simply removed the acronym as we do not refer to it anywhere in the manuscript.

4. Line 79: the phrase "...increase the comprehension of..." is awkward; rephrase.

   We changed "increase the comprehension of" to "investigate" (line 98).

5. *** The organization of the simulation results and comparisons to experiments is more confusing than it needs to be, in my opinion. I think the authors should re-organize so that results are presented as follows. First, the baseline dynamics without any manipulation should be shown, across the relevant range of Iapp values. Second, the effects of the M block and, separately, the HCN block, when applied to the baseline conditions, should be shown. Third, the authors should show what happens when cAMP is increased or activated, again over a range of Iapp. Finally, the authors should show what happens when M block and HCN block are applied after cAMP is increased. In all of these instances, the authors should make clear where (in parameter space) there are regular APs and where there are MMOs. This is related to Figure 6 but should come earlier and be presented in a more organized, systematic way.

   Thank you, we agree that a reorganization and a more systematic presentation of the results have made it easier to follow the model results. However, in contrast to the suggestion by the reviewer, we prefer – for each simulation – to directly confront simulations with and without elevated cAMP (e.g., because of GCS application). We have therefore organized the initial part of the results section as follows. We first present the baseline dynamics and the results obtained with CGS application (Fig. 2), quantifying how CGS widens the interval where MMOs are observed (line 68). We then discuss model simulations (in the new Fig. 4) under ZD, ZD/CGS, XE and XE/CGS administration for the same values of applied current used for the baseline and "pure" CGS scenarios shown in Fig. 2, thus facilitating a direct comparison of the results, in line with the reviewer's proposal. We elucidate what happens when blocking the HCN channel (ZD and ZD/CGS) by quantifying the variations of the range of applied current where the model shows MMOs (lines 91-93). Then, we discuss the effect of blocking the M currents (XE and XE/CGS), again specifying the interval where MMOs are obtained (lines 108-110). Finally, we show the results obtained for the discussion regarding the partial inhibition of the M channels using FSK (Fig. 5 and lines 117-124).

6. Line 106: I believe "leftward" should be replaced with "rightward".

   We used "leftward" and not "rightward" because we wanted to focus on the onset of the oscillatory phenomena, which happens at lower values of I_{App} for the red FF curve than for the black FF curve. We have now specified that the leftward shift regards the onset of the FF curve (line 132).

7. ** The authors introduce confusion by referring to (V,h,s) as the "fast subsystem" while also noting that h, s are slower than V and stating that (V,h,s) is itself a "slow-fast system". Perhaps they were skittish about explicitly referring to 3 timescales, but I think that would be the better way to go. So w, r will be superslow; h, s will be slow; and V will be fast. It will be necessary to come up with a name for the (V,h,s) system. I think that calling that the slow-fast subsystem would be fine. Or the authors could call it the layer system.

   We agree with this observation, and have changed "fast subsystem" to "slow-fast subsystem" each time we refer to the (V, h, s) subsystem of the 5D model. We also mention explicitly that there are three time scales with w and r being super-slow, e.g., in the Abstract and in the Introduction.

8. The equation where tilde(r) is introduced has a typo ($g_{HNC} \to g_{HCN}$). In the same line of the paper, more explanation is needed about how to interpret the Delta g terms.

   Thank you for noticing the typo, which has been corrected. A brief explanation of Δg_{HCN} and Δg_M has been added (lines 172-176) with a reference to the Materials and Methods for further details (see also the answer to point 19).

9. *** Related to the previous comment: I find the use of tilde(r) and tilde(w) confusing. Why can't the authors just vary w and r? If they are going to use tilde(w) and tilde(r), they should also state explicitly how they show up in the model equations. Currently, for example, the authors state above line 346 that they introduce scaling factors and define tilde(w) and tilde(r), but once these terms are defined, the authors do not state how the V equation is adjusted to include these terms.

   The way we modelled the action of cAMP (as an increase of HCN and M-channel conductances) means that we should use different bifurcation diagrams for situations without ($cAMP = 0$) and with ($cAMP = 1$) raised cAMP, if we wished to use r and w as bifurcation parameters. By rewriting the equations using $\tilde{r}$ and $\tilde{w}$ we, in a certain sense, have cAMP act on the gating variables (although $\tilde{r}$ and $\tilde{w}$ are no longer gating variables and could, in principle, be larger than one). This is a purely algebraic manipulation, but it has the advantage that the same bifurcation diagrams with $\tilde{r}$ and $\tilde{w}$ as parameters can be used both for scenarios without and with raised cAMP. Moreover, cAMP-mediated activation of one or both of HCN and/or M-type currents correspond to increases in $\tilde{r}$ and/or $\tilde{w}$, i.e., steps in the bifurcation diagrams, see e.g. Fig. 7B.

   We have rewritten the part where we introduce the scaled variables $\tilde{r}$ and $\tilde{w}$ substantially (lines 425-428 in Materials and Methods). We explicitly write how $\tilde{r}$ and $\tilde{w}$ enter the model when I_{HCN} and I_M are rewritten, see Eqs. (9)

and (10). Moreover, it is now explicitly mentioned that the 3D model in this formulation is independent of cAMP, which is completely incorporated in the super-slow variables $\tilde{r}$ and $\tilde{w}$, which are treated as parameters in the 3D model.

10. Lines 197-8: Provide more details about which aspects of Fig. 6 are explained here; "This explains the results shown in Fig 6..." is not adequate. For example, Fig. 6 shows results about both H and HCN activation via increased cAMP, which is not discussed in the earlier part of this paragraph.

    Lines 229-242: We have added additional explaination using $\tilde{r}$ and $\tilde{w}$ to understand the reason why the FF curve during XE and CGS stimulation shifts towards the left compared to the administration of XE only. In this case, since XE blocks the M current (Δg_M, g_M both zero), the V equation is no longer dependent on $\tilde{w}$. This results in analysing the dynamics along the $\tilde{r}$ axis of the 2P-BD, so that CGS application corresponds to a vertical step, which depending on I_{app} can move the system from the silent to the MMO region, or from the MMO to the LAO region.

11. Fig. 9: If Delta gM=0, then the elevated cAMP case is actual identical to the control case, so why don't the blue and magenta curves meet at Delta gM=0 (similary for red and black)? Is this because of HCN? Please clarify. Also, please mention in the caption what value of cAMP is used here.

    No, setting Δg_M equal to 0 does not make the models with raised cAMP equivalent to the control case. The discrepancy is because of the non-zero value of Δg_{HCN}, i.e., cAMP increases the HCN conductance even if it is assumed to have no effect on the M current ($\Delta g_M = 0$). We added the default parameters of g_{HCN} and Δg_{HCN} used to derive the two-parameter bifurcation diagram, and the values of $cAMP$ as requested, in the caption of the figure (Fig. 9).

12. Line 231: same issue with fast vs. slow-fast as mentioned above.

    We changed as explained at point 7 above.

13. Please add a new 3-d figure showing the surface C0 along with the folds L and the various S sheets.

    We added a new 3D figure (Fig. 10) showing the critical manifold $\mathcal{C}_0$, its attracting and repelling submanifolds, the fold, the unstable fixed point and the folded node. The simulated trajectory of the 3D system is superimposed on top of the critical manifold.

14. Line 263: You cannot assume that PLOS CB readers are familiar with canards.

    We rewrote this part slightly and now introduce canards when commenting on Fig. 12 (lines 320-324), briefly mentioning that these are special solutions that connect the attracting and repelling sheets of the slow manifold. We inserted a reference to the Multiple Time Scale Dynamical System section, where the reader can find additional information on canards, and we expanded this part (lines 440-453). Here, we make it explicit that we call *canards* all those "important" solutions that organize the folded node funnel, i.e., the secondary canards, for the sake of simplicity.

15. Please provide more information about Fig. 10 to make it clear why the CGS case with $I_{app} = 250$ gives 1 LAO per cycle, while $I_{app} = 300$ gives > 1 LAO.

    The difference with respect to the number of LAOS between $I_{app} = 250$ and $I_{app} = 300$ is due to differences in the return mechanisms. For $I_{app} = 250$ the orbit is sent back into the funnel after each LAO, whereas for $I_{app} = 300$ the orbit does not enter the funnel after the first LAO but only after the second LAO. To make this clearer, we have added the strong canard and the funnel region to the figure (now Fig. 11) and extended the text (lines 314-318).

16. Inset of Fig. 11, upper right: Why are there sharp corners on the trajectory?

    The upper right inset (now Fig. 12) presents the intersection between the attracting and repelling slow manifolds and a chosen plane; it is not a trajectory. The sharp corners are due to the implemented numerical procedure, where these manifolds are constructed from a grid of initial values and the system is solved forward/backward in time. However, the solutions are highly sensitive to the selected initial conditions, which results in the relative large distance between the points used to draw the curve in question, which therefore (misleadingly) seems to have sharp corners.

17. Fig. 11: Please tell us how the ξ_i are computed. Also, more explanation and labelling is needed so that readers can see that the trajectory is projected to the left of ξ_0 after the 1st return for $I_{app} = 300$.

    We now explain how the sheets of the critical manifold were computed and how canards were found at the very end of Material and Methods.

    The figure (now Fig. 12) was extended by color coding, along with starting and ending markers (circles and crosses, respectively), of the voltage trace and of the corresponding phase-plane trajectories to ease direct comparison. These additions hopefully make it clear how the different parts of the simulations are

related to the funnel, canards and the rotational sectors. The corresponding explanation in the main text has also been extended (lines 328-332).

18. *** Discussion: The authors should say more about the possible functional role of the MMOs that they discuss. They should also state some explicit predictions from their multiple timescale analysis.

    Thank you for these suggestions. We now discuss the physiological relevance of the control of neuronal firing frequency via MMOs by HCN and M channels in the Discussion (lines 360-365). We also make explicit predictions based on the 2-parameter BDs in Fig. 7 to illustrate how our analyses can provide insight (lines 375-385).

19. Methods: How can g_{HCN} and g_M be called "maximal" conductances when the Delta terms are positive?

    The parameters g_{HCN} and g_M are the maximal whole-cell conductances (the conductance obtained when all "activable" channels are open) at basal (low) cAMP, i.e., without activation of the cAMP-dependent pathway ($cAMP = 0$ in the equations). The Δ-terms model the increase in this maximal whole-cell conductance, which is how we decided to model the increase in cAMP in agreement with Lezmy et al. (Ref. 4) and Schroeder et al. (Ref. 24). Biophysically, this could correspond to an increase in the number of "activable" channels ("waking up" dormant channels or insertion of more channels in the membrane) or an increase in the single-channel conductance. We now added a few sentences to clarify this concept (after the ODE system (1)–(8)).

20. Table 1: How can you have two different temperatures for 2 different current gates in the same model of the same cell? Also, why is E_L specified to the hundredths place while none of the other parameters are? Is the model extremely sensitive to E_L? Finally, why is E_L so negative? This is an unusually low value, in my experience.

    The parameters $T_{ref,1}$ and $T_{ref,2}$ are not temperatures ($T = 37°C$ is the temperature of the experiment), but reference parameters that describe the temperature-dependence of the fast Na^+, slow K^+ and HCN channels as specified in the caption of Table 1, basically scaling factors of ϕ_p – and hence of α_p and β_p. The model is rather sensitive to E_L, but we checked that the conclusions of the paper also hold when the value of E_L is changed moderately (by up to ∼5 mV). The used value is equal to the one used in [4], which, in turn, agrees with the value in the original model in [7].

21. Model reduction: The authors should provide a quantitative justification for the statement that h, s are slower than V.

    At the beginning of the section Model Reduction, we now provide statements regarding the timescale separations between V and (h, s), and between s and (r, w) to justify the claims of V being fast, (h, s) slow, and (r, w) super-slow.

22. Pg. 17, top: The authors should provide some justification for the values that they choose for T, t0, T-t0.

    After Table 1: We have added a few sentences to justify the chosen values.

I look forward to seeing the revisions! I certainly hope that the authors can make these changes and improvements. I would love to use a published version of this work in my graduate mathematical neuroscience course.

We hope that the reviewer appreciate the changes we have made, and we are flattered that she/he considers using our work in her/his course! Waiting for the published version, the reviewer might want to use the preprint available at `https://doi.org/10.1101/2023.10.02.560623`.
